# Administration of an Immune Stimulant during the Transition Period Improved Lipid Metabolism and Rumination without Affecting Inflammatory Status

**DOI:** 10.3390/ani9090619

**Published:** 2019-08-28

**Authors:** Matteo Mezzetti, Andrea Minuti, Fiorenzo Piccioli-Cappelli, Gianfranco Gabai, Erminio Trevisi

**Affiliations:** 1 Department of Animal Sciences, Food and Nutrition, Faculty of Agriculture, Food and Environmental Science, Università Cattolica del Sacro Cuore, 29122 Piacenza, Italy; 2 Department of Comparative Biomedicine and Food Science, University of Padova, 35020 Legnaro (PD), Italy

**Keywords:** immune stimulant, metabolic disorders, transition period, immune dysfunctions

## Abstract

**Simple Summary:**

Immune stimulants are widely used to address immune dysfunctions that occur in transitioning dairy cows, reducing the likelihood they will develop infectious diseases. This study elucidates the effectiveness of an immune stimulant in promoting rumination recovery, reducing lipid mobilization and ketogenesis, and affecting the levels of circulating antioxidant systems in early lactation. These findings highlight the stimulant’s potential effect in treating metabolic disorders of the transition period in dairy cows.

**Abstract:**

Omnigen-AF (OAF) increases leukocyte functions in immunosuppressed animal models and reduces incidence of infectious diseases in early lactating dairy cows, although its mode of action is still unclear. This study aims to provide a wider perspective of the metabolic effect of OAF to test its potential as a strategy to address metabolic disorders of the transition period. A group of 10 Holstein dairy cows were divided into 2 groups: The treated group (IMS; 5 cows) received 32.5 g of OAF twice a day (65 g d^−1^) as top-dress in the morning and afternoon feeds from −55 to 42 days from calving (DFC), whereas the control group (CTR; 5 cows) received no supplementation. From −62 to 42 DFC, body condition score, body weight, dry matter intake, rumination time and milk yield were measured; blood samples were collected weekly to assess a wide hematochemical profile and to test white blood cell functions by *ex-vivo* challenge assays. At 30 DFC, rumen fluid was collected and analyzed for pH, volatile fatty acids composition, urea nitrogen, and lactate contents. Data were submitted to ANOVA using a mixed model for repeated measures, including treatment, time, and their interaction as fixed effects. OAF decreased blood nonesterified fatty acids and beta hydroxybutyrate concentrations and increased rumination time in early lactation. Leukocytes from IMS cows had lower lactate production and lower glucose consumption after *ex-vivo* stimulation. OAF did not reduce the acute phase response indicators and reduced the blood concentrations of albumin and antioxidants after calving, suggesting impairment of hepatic functions related to protein synthesis and antioxidant management. Nevertheless, the lack of effect on bilirubin and liver enzymes refutes the possibility of severe liver damage occurring with OAF supplementation. Positive effects in reducing mobilization of body fats and ketogenesis and in increasing rumination time after calving suggest OAF effectiveness in preventing metabolic disorders of the transition period.

## 1. Introduction

The immune system eliminates sources of tissue injury, restores immune homeostasis, and returns tissues to their normal function [1]. Innate immunity defends the organism until lymphocytes develop antibodies against antigens [2]. During inflammation, polymorphonuclear cells (PMN) migrate with blood flow to peripheral tissues, starting the production of antibacterial reactive oxygen metabolites (ROM) through the respiratory burst process [3,4].

In PMNs of dairy cows, functions related to ROM production, myeloperoxidase activity, chemotaxis, and phagocytosis are impaired from 2–4 weeks prior through to approximately 2 weeks after parturition [5,6,7]. Consequently, cows become hyposensitive to antigens, and the incidence of infectious diseases increases [8,9]. From the early dry period to the first month of lactation, the immune system becomes unable to resolve inflammation [10,11], triggering chronic phenomena [7,12,13]. The activation of leukocytes during chronic inflammation in combination with increased metabolic activity during the transition period could contribute to the production of oxidant species, depleting antioxidant defenses, and triggering oxidative stress [1,14,15]. Those conditions increase the development of metabolic disorders in the transition period [16,17].

The immune stimulant Omnigen-AF (OAF) contains a mixture of active dried Saccharomyces cerevisiae, dried Trichoderma longibrachiatum fermentation product, niacin, vitamin B12, riboflavin-5-phosphate, d-calcium pantothenate, choline chloride, biotin, thiamine monohydrate, pyridoxine hydrochloride, menodione dimethylpyrimidinol bisulfate, folic acid, calcium aluminosilicate, sodium aluminosilicate, diatomaceous earth, calcium carbonate, rice hulls, and mineral oil. It improves PMN functions in immunosuppressed goats that receive dexamethasone treatments [18], altering mRNA transcripts and protein expression related to the innate immune system [19,20]. Its mode of action has not been fully elucidated, but interactions between the yeast and fungal cell walls that compose OAF and the intestinal M-cells, mediating the response of lymphoid tissue, is a hypothetical mechanism [21]. Its administration in transition dairy cows down-regulated the expression of genes responsible for leukocyte death, inhibiting apoptosis and increasing the number of circulating leukocytes [6]. mRNAs encoding for cytokines were up-regulated, improving sensitivity of PMNs to extracellular signaling and cell-to-cell communication [18]. OAF also enhanced PMN diapedesis through the up-regulation of mRNA encoding for L-selectin [22] and augmented phagocytosis and respiratory burst by improving the oxidative phosphorylation pathway involved in ROM production [6,20,23]. Improved PMN functions account for the reduced incidence of mastitis found with OAF supplementation [6], suggesting its use as a potential strategy to reduce the incidence of infectious diseases in the transition period. However, no previous studies have investigated the effect of OAF on the sudden metabolic changes seen in the transition period, although elucidating such an effect is crucial in assessing the effectiveness of OAF in preventing metabolic disorders. Thus, the objective of our study was to assess the effect of supplemental OAF from dry-off to early lactation on metabolism, systemic inflammatory status, oxidative status, and immune status in dairy cows. We hypothesized that metabolic status around calving could be improved by OAF, mitigating systemic inflammation and oxidative status of treated animals.

## 2. Materials and Methods

The trial was carried out at the Università Cattolica del Sacro Cuore research dairy barn (Cerzoo Experiment Station, San Bonico, Piacenza, Italy) in accordance with Italian laws on animal experimentation (DL n. 116, 27/01/1992) and ethics (authorization of the Italian Ministry of Health N 65427; 29/10/2010-PR).

### 2.1. Experimental Design and Animal Management

A group of 10 Italian Holstein dairy cows (number of lactations: 1.9 ± 1.1; milk yield in the last lactation: 11547.8 ± 2576 kg; average lactation length: 353.1 ± 54 days [mean ± SD]) were housed in individual tied stalls under controlled environmental conditions (room temperature of 20 °C, relative humidity of 65%, 14 h of light) from −62 to 42 days from calving (DFC). At −55 DFC cows were dried off and treated with a mammary antibiotic (Mamyzin A; Haupt Pharma Latina S.r.l., Borgo San Michele, Latina, Italy). Before dry-off and after calving, cows were milked twice a day at the stand at 4:00 am and 4:00 pm. All cows were individually fed a component diet of 2 equal meals of forages at 12-h intervals and 2–8 meals of concentrates supplied by computer feeder. Residuals were individually weighted after each meal, and the amount of DM offered was changed based on daily feed consumption. Before dry-off, animals received a lactating ration with soybean meal, alfalfa dehydrated hay, and corn silage (Phase 1). After dry-off, animals received only hay until −48 DFC. From −47 DFC to −7 DFC animals received a hay-based ration with soybean meal and corn silage (Phase 2). Seven days before the expected day of calving, 1 kg of lactation concentrate was gradually added to the diet (Phase 3). After calving the diet was enriched with 3 kg of alfalfa-dehydrated hay, and 2 kg week^−1^ of corn silage was gradually added to the diet to a maximum of 20 kg d^−1^. Grass hay was gradually reduced to 2–2.5 kg d^−1^, and concentrate was increased by 0.5 kg d^−1^ to meet the requirement of 1 kg per 3 kg of milk produced (Phases 4 and 5). The same batches of hay and corn silage were used during the trial. Feeds were collected fortnightly; after dry matter determination, samples were pooled for subsequent analyses. Feeds and diet composition are shown in Table 1.

**Figure 1:**
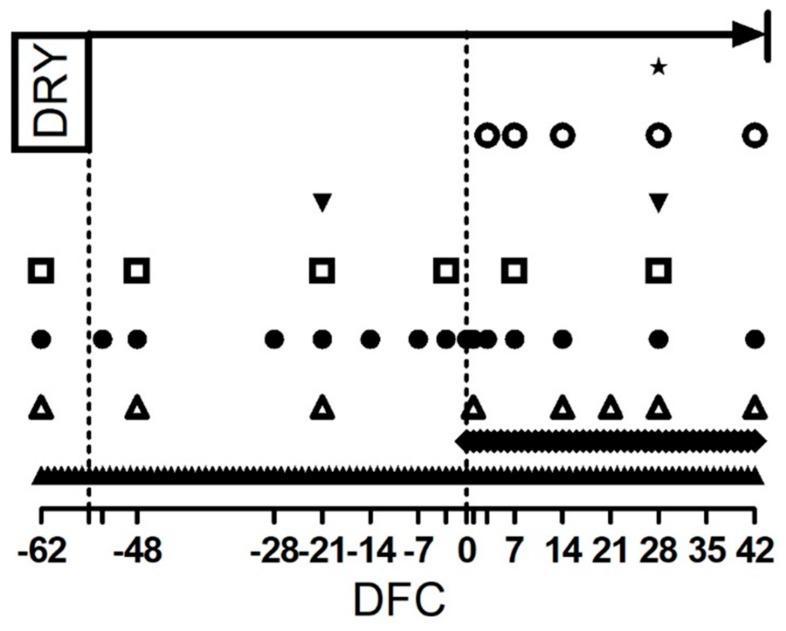
Scheduled time points, expressed as day from calving (DFC), for dry-off (DRY), treatment administration (upper arrow), rumen sample collection (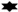), milk sample collection (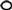), and blood sample collection for the interferon gamma release assay (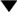), carrageenan skin test performance and blood cell profile and whole blood stimulation assay (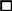). Blood sample collection for metabolic profile (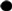). Milk yield measurement (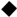), body weight and body condition score determination (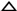), and dry matter intake and rumination time determination (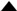). Empty ticks indicate −55, −53, −3, 1 and 3 DFC, respectively.

At dry-off, cows were divided into 2 homogeneous groups by body condition score (BCS), body weight (BW), number of lactations, milk yield, and age. The group treated with immune stimulant (IMS; 5 cows) received 32.5 g of Omnigen-AF^®^ (Phibro Animal Health Corporation, Teaneck, NJ, USA) twice a day (65 g d^−1^) as top-dress on the morning and afternoon corn silage distribution, whereas the control group (CTR; 5 cows) received no supplementation. The amount of immune stimulant was determined on the basis of the average BW of the animals at dry-off to ensure the daily consumption of 9 g of product per 100 kg of BW was met. The daily consumption of the whole OAF dose provided was assessed by an operator after each meal. Between −62 and 42 DFC, periodic checks were performed, and blood samples were collected regularly according to the time schedule shown in Figure 1 and described in the following sections.

### 2.2. Body Weight, Body Condition Score, Dry Matter Intake, Rumination Time, and Milk Yield

The BW was measured in the morning, before feeding administration, with a single walk-in scale. BCS was determined by the same operator with a 1 to 4 scale [24], and its variation (∆BCS) was calculated as the difference between the value at calving day and at 28 DFC. The daily individual feed intake was measured by weighing the amount of feed administered and their refusals, and calculating the difference between them. The composition of refusal was also evaluated daily, and the DMI was calculated based on the DM content of each feed. Rumination time was registered using the Ruminact system (SCR Europe, Podenzano, PC, Italy), and data were processed according to [25]. Milk yield was weighed after each milking. Daily values of DMI, rumination time and milk yield were used to create an average weekly value.

### 2.3. Health Status

Health conditions of cows were monitored daily from −62 to 42 DFC. Body temperature was measured daily with a rumen bolus (DVM System TempTrack™, HerdStrong, LLC, Greeley, CO, USA). Mastitis was diagnosed by visual evaluation of abnormal milk from each quarter and by somatic cell count (SCC) analysis in suspicious cases. Retained placenta was diagnosed when the fetal membranes were not expelled within 24 h after calving. Endometritis and metritis were diagnosed according to [26]. Milk fever, displacement of abomasum, and pneumonia were diagnosed by examination by a veterinary practitioner. Diarrhea was diagnosed by visual evaluation of feces consistency and color according to the fecal score method [27], assuming diarrheic feces to have a fecal score ≤2. Sub-clinical ketosis was retrospectively diagnosed based on blood levels of beta-hydroxybutyrate (BHB), assuming a threshold of 1.4 mMol/L as a cut-off point [28].

### 2.4. Blood Sample Collection

Blood samples were harvested trough jugular venipuncture in evacuated collection tubes (BD Vacutainer; BD and Co., Franklin Lakes, NJ, USA) before the morning feeding. Samples were used for different assays (Figure 1) and processed according with the following sections. After all blood samples were collected, all frozen samples were thawed and analyzed (maximum storage time of 6 months).

#### 2.4.1. Metabolic Profile Assessment

Samples were collected into heparinized tubes and processed as described by [29]. After processing, plasma samples were stored at −20 °C. A clinical auto-analyzer (ILAB-650, Instrumentation Laboratory, Lexington, MA, USA) was used to determine the concentration of glucose, nonesterified fatty acids (NEFA), BHB, urea, creatinine, Ca, P, Mg, Na, K, Cl, Zn, aspartate amino transferase-glutamate oxaloacetate transaminase (AST-GOT), gamma glutamyl transferase (GGT), alkaline phosphatase (ALP), total protein, haptoglobin, ceruloplasmin, albumin, total bilirubin, cholesterol, and globulin in accordance with [29]. Furthermore, ROM, ferric-reducing antioxidant power (FRAP), nitrate (NO_3_), nitrite (NO_2_), and nitric oxides (NOx) were determined according to [30], paraoxonase (PON) according to [12], thiol groups (SHp) according to [31], myeloperoxidase according to [32], and advanced oxidation protein products (AOPP) according to [33]. The ROM/FRAP ratio was also calculated to provide an integrated oxidant status index according to current recommended practices [34]. Finally, L-lactic acid (LLA) and D-lactic acid (DLA) were determined with a commercial kit (K-DLATE, Megazyme Co., Wicklow, Ireland).

Calibration was performed through commercial standards for Na, K, Cl, Zn, ceruloplasmin, albumin, protein, bilirubin, ALP, NEFA, BHB, ROMt, FRAP, DLA, LLA, and nitrogen species. Calibration for remaining indicators was performed through internal standards. Four different quality controls were used to test the repeatability and precision for each parameter during each assay. A multi-detection microplate reader (BioTek Synergy 2, Winooski, VT, USA) and commercial kits for ELISA were used to determine the concentration of IL-1 beta (IL-1B; ESS0029; Thermo Scientific, Frederick, MD, USA) and IL-6 (ESS0027; Thermo Scientific) according to [7] and those of serum amyloid alpha (SAA; TP-802, Tridelta D.L., Ireland). Oxygen-reactive antioxidant capacity (ORAC) was determined with a fluorimetric method according to [30]. Retinol, tocopherol, and beta-carotene were analyzed by reverse-phase HPLC (LC-4000, Jasco Europe, Carpi MO, Italy), as described by [7]. Further details on the analytical procedures adopted in blood analysis are reported in Supplementary File 1.

#### 2.4.2. Blood Cell Profile

Samples were collected in K-EDTA tubes, kept at 4 °C after collection and analyzed within 4 h of collection with Cell-DYN 3700 (Abbott Diagnostic Division, Santa Clara, CA, USA). A laser optic assay was used to determine the total leukocytes, neutrophils, lymphocytes, monocytes, eosinophils and basophils. The neutrophil to lymphocyte ratio was calculated. The amount of red blood cells, hematocrit, mean cell volume, red cell distribution width, number of platelets, and mean platelet volume were determined via electrical impedance assay. The amount of hemoglobin, mean cell hemoglobin, and mean cell hemoglobin concentration were determined using spectrophotometric assay.

#### 2.4.3. Whole Blood Stimulation Assay

Samples were collected in heparinized serum tubes and processed according to [7]. Briefly, whole blood was freshly stimulated with 0 (baseline), 0.01 (low dose), and 5 µg/mL (high dose) of bacterial lipopolysaccharides (Escherichia coli O111:B4; Sigma–Aldrich Company Ltd., Gillingham, UK, Cat. No. L3012). After stimulation, all samples were positioned on a rotator within a heated incubator (Grant Boekel, HIR10 M) set to a temperature of 38 °C and a rotation speed of 3 times/min for 3.5 h. Plasma was collected after centrifugation at 8500× *g* for 16 min at 4 °C. After whole blood stimulation assay (WBA), plasma samples were stored at −80 °C for measurement of glucose, DLA, LLA, IL-1B, IL-6, NOx, NO_2_, and NO_3_. Variation of plasma indicators after WBA with L and H doses of LPS were expressed as fold change relative to the baseline.

#### 2.4.4. Interferon Gamma Release Assay

For the interferon gamma (IFNG) release assay, whole blood samples again were collected into heparinized tubes (Figure 1). After collection, the tubes were stored in a vertical position in a warm bath at a temperature of 38 °C and transported to the laboratory within 20 min for the stimulation procedure. Whole blood was used in an IFNG release assay after Mycobacterium avium stimulation (internal method IZSLER, MP 13/011). Briefly, two 1-mL aliquots of each blood sample were distributed in a 24-well tissue culture microtiter plate. One well was supplemented with 100 µL of a 1:10 dilution of Mycobacterium avium purified protein derivative (PPD, IZS Umbria e Marche, Perugia, Italy, 0.5 mg/mL) and PBS, and 1 well contained 100 µL of sterile PBS as control. The plate was positioned in a heated incubator (Grant Boekel, HIR10 M) set to a temperature of 38 °C with a relative humidity of 95% for 24 h. After incubation, the blood was centrifuged at 8500× *g* for 16 min at 4 °C, and plasma was stored at −20 °C until use. Plasma was later thawed and analyzed in a sandwich ELISA for bovine IFNG with two monoclonal antibodies, as previously described [35]. Results were evaluated in terms of optical density difference (∆OD) between avian PPD-stimulated and control wells.

### 2.5. Milk Sample Collection and Analysis

Milk samples were collected into 100-mL polypropylene bottles (International Scientific Supplies Ltd., Bradford, UK) during the morning milking (Figure 1). Butterfat, protein, lactose, casein content, titratable acidity, and coagulation properties (rennet clotting time [rCT] and curd firmness at 30 min [a30]) were measured by using infrared instrumentation (MilkoScan FT 120, Foss Electric, Hillerød, Denmark) according to [36] and [37]. The output of fat and protein and the fat to protein ratio were also calculated. Urea nitrogen in skimmed milk was determined by a spectrophotometric assay, using a urea nitrogen kit (cat# 0018255440, Instrumentation laboratory, Milano, Italy) in association with a clinical auto-analyzer (ILAB-650, Instrumentation Laboratory, Lexington, MA, USA). SCC were determined using an optical fluorimetric method with an automated cell counter (Fossomatic 180, Foss Electric).

### 2.6. Carrageenan Skin Test

The carrageenan skin test (CST) was performed as specified by [7] to evaluate peripheral immune response (Figure 1). Skin thickness was measured using a skinfold caliper (cat# 470119-588, VWR, Radnor, PA, USA) immediately before carrageenan injection (day 0) and again at 2 and 9 days after the injection. The total response of each challenge was calculated as the area under the curve of the thickness measured at day 2 and day 9, subtracting the thickness measured at day 0.

### 2.7. Rumen Fluid Measurements

Rumen samples were collected with an orogastric probe (Ruminator, Proofs Products, Guelph, ON, Canada) before the morning feed administration (Figure 1). To reduce the buffer effect of saliva, the first 0.5 L of rumen juice was discarded. The pH was immediately measured (GLP 21, Crison Instrument SA, Alella, Barcellona, Spain, ESP). A 2-mL aliquot of the supernatant was transferred into tubes containing 1 mL of 0.12 M oxalic acid and immediately frozen at −20 °C for later analysis. Total VFA concentration and molar proportion of acetic, propionic, butyric, iso-butyric, valeric, iso-valeric, caproic, iso-caproic, and enanthic acids were analyzed as previously described [31]. Single VFA were expressed as relative amounts compared with total VFA concentration. A spectrophotometric clinical auto-analyzer (ILAB-650, Instrumentation Laboratory, Lexington, MA, USA) and commercial kits for urea nitrogen (cat# 0018255440, Instrumentation laboratory, Milano, Italy) and lactate (K-DLATE, Megazyme Co., Wicklow, Ireland) were used to assess the concentration of ammonia-N and of LLA, DLA and total lactic acid, respectively.

### 2.8. Statistical Analysis

A limitation of our study was the small sample size; thus, a post hoc power analysis considering an alpha value of 0.05 was run on the measurements included in current results. High power (1−β > 0.8) was found for IL1B, IL6, cholesterol, Zn, Albumin, PON, FRAP, AOPP, tocopherol, thiol groups, myeloperoxidase, and NEFA. The remaining measurements had a moderate power (0.5 < 1−β < 0.4). Data were analyzed in SAS software, version 9.4 (SAS Inst. Inc., Cary, NC, USA) and are presented in the graphs and tables as means and pooled standard error for individual means of treatments over time. Before analysis, normality of data distribution was verified for each parameter by evaluating skewness and kurtosis according to the Shapiro test in SAS. Non-normally distributed parameters were normalized through natural logarithms (for plasma measurements: IL-1B, IL-6, LLA, GGT, bilirubin, beta-carotene, NOx, NO_2_, and NO_3_; for blood cell profile: eosinophils; for WBA: the fold change of IL-1B, IL-6, glucose, DLA, LLA, NO_2_, NO_3_, and NOx; the total response to CST; and for milk quality measurements: fat, fat output, fat/protein ratio, rCT and SCC) or square root transformation (for plasma measurements, the BHB) and back transformed to be plotted in the graphs and tables.

Data on DMI, BW, BCS, rumination time, milk yield, plasma measurements, blood cell profile, WBA, milk quality measurements, response to IFNG release assay, and CST underwent ANOVA using a mixed model for repeated measures (Mixed procedure, SAS Inst. Inc., Cary, NC, USA) in accordance with [38]. The statistical model included the fixed effect of treatment (TRT; CTR and IMS), time (t) and their interaction (TRT × t).

y_ijk_ = µ + α_i_ +δ_ij_ + τ_k_ +(ατ)_ik_ + e_ijk_

where y_ijk_ is the response at time k on animal j in treatment group i, µ is the overall mean, α_i_ is a fixed effect of treatment i, δ_ij_ is a random effect of animal j in treatment group i, τ_k_ is a fixed effect of time k, (ατ)_ik_ is a fixed interaction effect of treatment i with time k, and e_ijk_ is random error at time k on animal j in treatment i. For parameters that were measured daily (DMI, rumination time, and milk yield), the time effect considered the average weekly value; for other parameters (BW, BCS, plasma measurements, blood cell profile, WBA, milk quality measurements, IFNG release assay, and CST), it considered single DFC. The time was considered as a repeated measure within the cow, and the cow was assumed as a random effect. For WBA, the dose (D; low and high) and the full interaction effect (TRT × t × D) also were considered. The analysis was carried out using three covariance structures: autoregressive order, compound symmetry, and spatial power with their heterogeneous counterparts. When a heterogeneous covariance structure was chosen, the bars errors for each time point have been provided in the figures. These were ranked according to their Akaike information criterion, with the structure that had the lowest Akaike information criterion being chosen [38]. A preliminary analysis was conducted, and all parameters were covariate using data collected at −62 DFC as the baseline. The covariate was included in the final model only for parameters that had a significant covariate effect in the preliminary analysis, adopting *p* ≤ 0.1 as a cutoff for covariate inclusion (among plasma measurements: glucose, IL-1B, IL-6, cholesterol, retinol, and tocopherol).

Data on rumen fluid measurements were analyzed by the same procedure (Mixed procedure, SAS Inst. Inc., Cary, NC, USA), considering only the fixed effect of TRT.

After the analysis, the residuals were plotted to assess for model assumptions of normality and homoscedasticity. The post-hoc comparison between treatments was done using the F-test and are discussed when the *p*-value for main effect was *p* ≤0.05. Main effects at *p* ≤ 0.10 are discussed in the context of tendencies. Differences between treatments at single time points are discussed at *p* ≤ 0.10 for the main interaction effect.

## 3. Results

The time effect was highly significant (*p* < 0.01) for most of the parameters included in the study. Thus, the time effect will not be presented in the Results section.

### 3.1. Body Weight, Body Condition Score, Dry Matter Intake, Rumination Time, and Milk Yield

**Figure 2:**
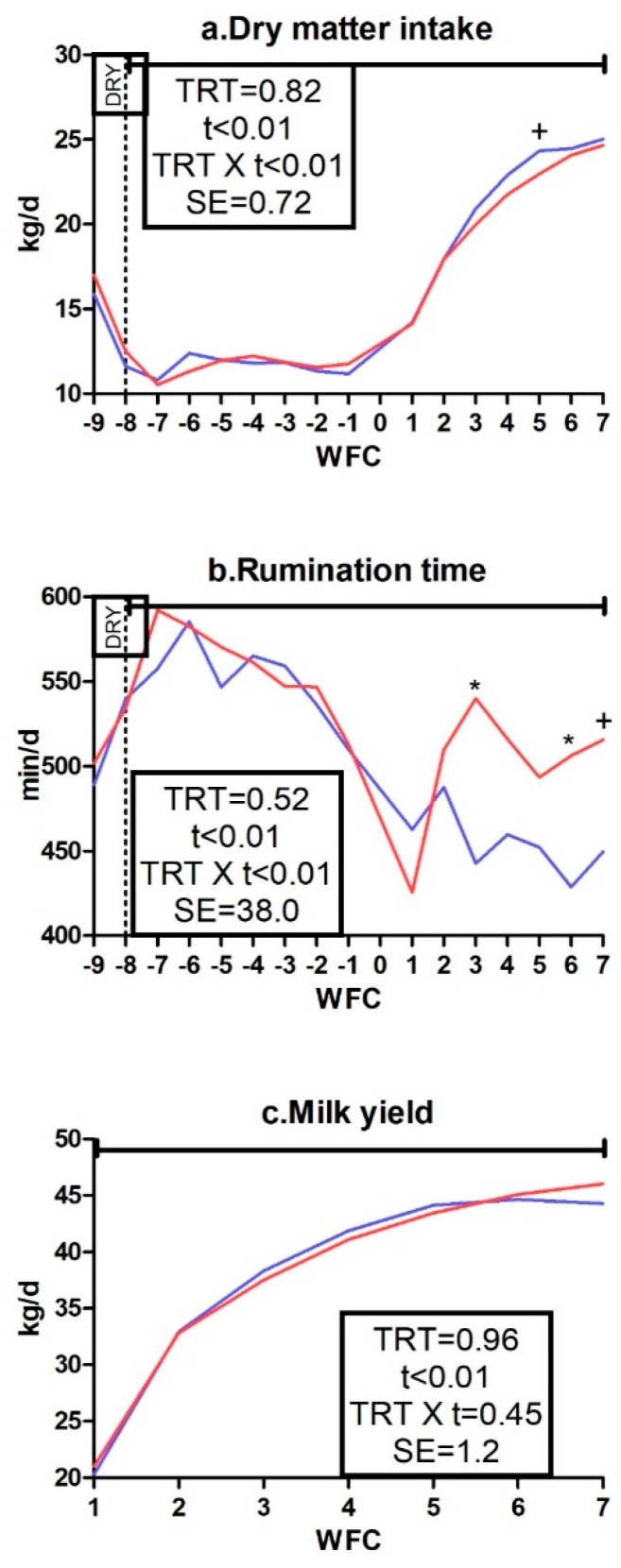
Average week values of dry matter intake (**a**), rumination time (**b**), and milk yield (**c**) in control dairy cows (CTR; blue line, n = 5) and cows receiving 65 g*d^−1^ of Omnigen-AF^®^ as top-dress (IMS; red line, n = 5) between −55 and 42 days from calving. Upper solid line indicates timing of treatment administration. TRT is treatment effect; t is time effect; TRT × t is the treatment × time interaction effect (+ is *p* < 0.10). WFC is weeks from calving; DRY is dry-off day (−55 days from expected calving). SE is standard error.

No TRT effect appeared for DMI, rumination time or milk yield (Figure 2a–c), but a TRT × t interaction appeared for DMI and rumination time (*p* < 0.01). The DMI tended to be lower in the IMS group than in the CTR group at 5 weeks from calving (*p* < 0.10). Rumination time was higher in the IMS group than in the CTR group at 3 and 6 weeks from calving (*p* < 0.05) and tended to be higher at 7 weeks from calving (*p* < 0.10). No effect was detected on body weight or BCS (Supplementary File 2).

### 3.2. Milk Quality and Rumen Fluid Measurements

Among milk quality and rheological measurements (Table 2), lactose had a significant TRT × t interaction (*p* = 0.03) and tended to be higher in the IMS group than in the CTR group at 7 DFC (*p* < 0.10). No effect appeared for the other milk measurements or rumen fluid measurements (Table 3).

### 3.3. Metabolic Profile

#### 3.3.1. Packed Cell Volume, Energy, Protein, and Mineral Metabolism Biomarkers

**Figure 3:**
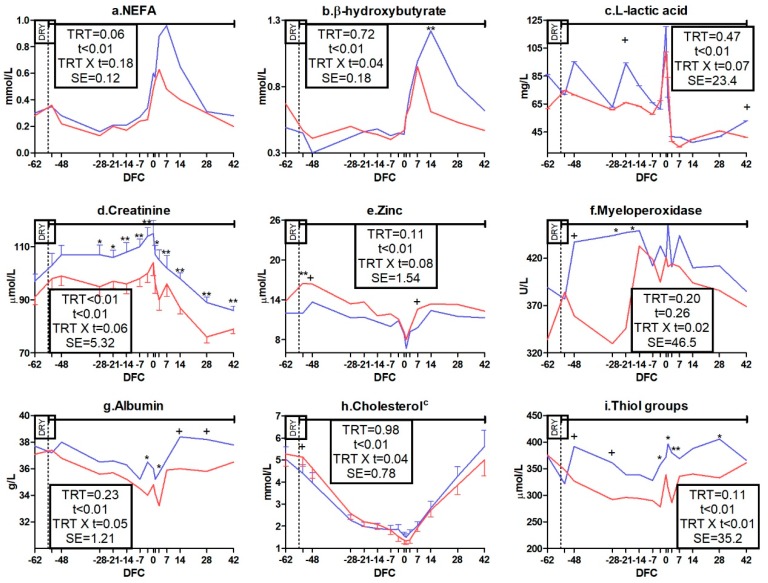
Time course of plasma concentrations of non-esterified fatty acids (NEFA; (**a**)), β-hydroxybutyrate (**b**), L-lactic acid (**c**), creatinine (**d**), zinc (**e**), myeloperoxidase (**f**), albumin (**g**), cholesterol (**h**), and thiol groups (**i**) in control dairy cows (CTR; blue line, n = 5) and cows receiving 65 g*d^−1^ of Omnigen-AF^®^ as top-dress (IMS; red line, n = 5) between −55 and 42 days from calving. Upper solid line indicates timing of treatment administration. TRT is treatment effect; t is time effect; TRT × t is the treatment × time interaction effect (** is *p* < 0.01; * is *p* < 0.05; + is *p* < 0.10). DFC is days from calving; DRY is dry-off day (−55 days from expected calving); SE is standard error; ^c^ Parameter was covariate on −62 value.

Among energy metabolism biomarkers, IMS cows tended to have lower concentration of NEFA (*p* = 0.06; Figure 3a). A significant TRT X t interaction was detected for BHB (*p* < 0.04; Figure 3b) and the interaction for LLA trended toward significance (*p* = 0.07; Figure 3c). In comparison to CTR cows, IMS cows had a lower BHB concentration at 14 DFC *(p* < 0.01) and tended to have a lower LLA concentration at −21 and 42 DFC (*p* < 0.10). No effect was detected on the packed cell volume or on glucose and DLA concentrations (Supplementary File 3a–c).

Among protein metabolism biomarkers, creatinine was affected by TRT (*p* < 0.01) and the TRT × t interaction trended toward significance (*p* = 0.06; Figure 3d), resulting in a lower concentration in IMS cows than in the CTR group between −28 and −3 DFC and from 7 DFC to the end of the experimental period (*p* < 0.05). No effect was detected on urea concentration (Supplementary File 3d).

Among mineral metabolism biomarkers, the TRT × t interaction for Zn trended toward significance (*p* = 0.08; Figure 3e). Its concentration was higher in the IMS group than in the CTR group at −53 DFC (*p* < 0.01) and tended to be higher at −21 and 42 DFC (*p* < 0.10). No effect was detected on the concentration of other minerals (Supplementary File 3e–j).

#### 3.3.2. Liver Function and Inflammation Biomarkers

No effect was detected on liver enzyme biomarkers (Supplementary Files 3k–l and 4a–b). Among inflammation biomarkers, globulin and total proteins were not affected (Supplementary File 5a–b), whereas myeloperoxidase experienced a TRT × t interaction effect (*p* = 0.02; Figure 3f). Cows treated with OAF had lower concentration of this enzyme at −21 and −14 DFC (*p* < 0.05). None of the positive acute phase proteins (APP) were affected by OAF (Supplementary File 4e–g). Among negative APP, a TRT × t interaction appeared for albumin (*p* = 0.05, Figure 3g) and cholesterol (*p* = 0.04; Figure 3h). The albumin concentration was lower in the IMS group than in the CTR group at −3 and 3 DFC (*p* < 0.05) and tended to be lower at 14 and 28 DFC (*p* < 0.10); in contrast, cholesterol concentration tended to be higher in the IMS group than in the CTR group at −53 DFC (*p* < 0.10). Concentrations of retinol and PON (Supplementary File 4h–i) as well as those of IL-1B and IL-6 (Supplementary File 4c–d) were not affected by OAF.

#### 3.3.3. Oxidative Status Biomarkers

**Figure 4:**
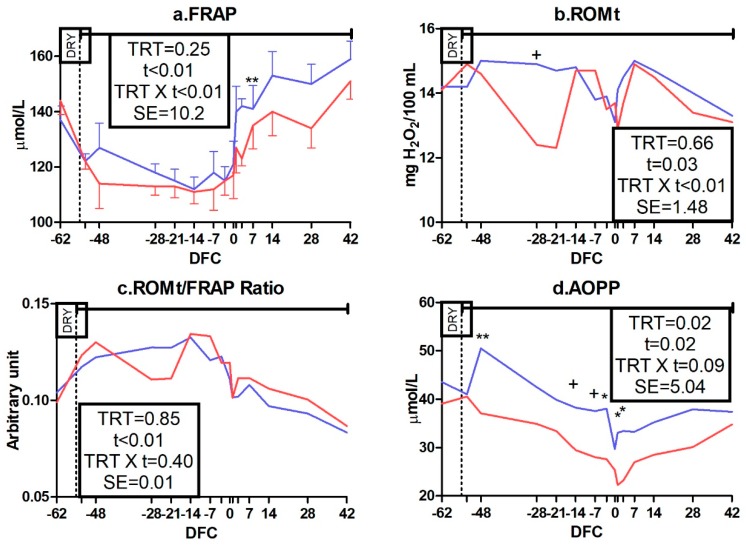
Time course of plasma concentrations of ferric-reducing antioxidant power (FRAP; (**a**)), total reactive oxygen metabolites (ROMt; (**b**)), reactive oxygen metabolites to ferric-reducing antioxidant power ratio (ROMt/FRAP ratio; (**c**)) and advanced oxidation of proteins product (AOPP; (**d**)) in control dairy cows (CTR; blue line, n = 5) and cows receiving 65 g*d^−1^ of Omnigen-AF^®^ as top-dress (IMS; red line, n = 5) between −55 and 42 days from calving. Upper solid line indicates timing of treatment administration. TRT is treatment effect; t is time effect; TRT × t is the treatment × time interaction effect (** is *p* < 0.01; * is *p* < 0.05; + is *p* < 0.10). DFC is days from calving; DRY is dry-off day (−55 days from expected calving); SE is standard error.

Among antioxidant capacity indicators, SHp and FRAP had a TRT × t interaction (*p* < 0.01; Figure 3i and Figure 4a). Compared to the CTR group, IMS cows had a lower concentration of SHp from −3 to 28 DFC (*p* < 0.05) and a lower concentration of FRAP at 3 DFC (*p* < 0.01). No effect was detected for tocopherol, beta-carotene, or ORAC concentration (Supplementary File 5c–e).

Among oxidant species, ROM had a TRT × t interaction (*p* < 0.01; Figure 4b) and tended to be lower in the IMS group than in the CTR group at −28 DFC (*p* < 0.10), whereas nitrogen species were not affected (Supplementary File 5f–h). The ROMt/FRAP ratio was not affected (Figure 4c) whereas AOPP was affected by TRT (*p* = 0.02) and tended to experience a TRT × t interaction effect (*p* = 0.09; Figure 4d). AOPP was lower in the IMS group than in the CTR group at −48, −3, 1, and 3 DFC (*p* < 0.05).

### 3.4. Blood Cell Profile

In the blood cell profile (Table 4), the mean cell volume and mean cell hemoglobin tended to be lower in IMS cows than in the CTR group (*p* = 0.08). The number of platelets tended to be higher and the mean platelet volume was lower in the IMS group than in the CTR group (*p* = 0.07 and = 0.01, respectively). A TRT × t interaction also appeared for mean platelet volume (*p* < 0.01): In IMS cows, volume was lower at −48 and −1 (*p* < 0.05) and tended to be lower at 7 DFC (*p* < 0.10) in comparison to CTR cows. No differences appeared for total leukocytes, neutrophils, lymphocytes, and hemoglobin (Table 4), and neither for neutrophil to lymphocyte ratio, eosinophils, red blood cells, mean cell hemoglobin concentration, or red blood cell distribution width (data not shown).

### 3.5. Whole Blood Stimulation Assay, Interferon Gamma Release Assay, and Carrageenan Skin Test

OAF treatment did not affect the cytokine response in WBA (Table 5). A tendency for a TRT × t interaction appeared for fold changes of glucose, DLA, and LLA (*p* = 0.06, 0.08 and 0.10, respectively; Table 6). The fold change of glucose tended to be higher in the IMS group than in the CTR group at −21 DFC (*p* < 0.10) and was higher at 7 DFC (*p* < 0.01). The fold change of DLA (*p* < 0.10) and that of LLA were lower (*p* < 0.05) in IMS cows than in the CTR group at 28 DFC. The response to LPS stimulation of nitrogen metabolites (Table 7), the response to IFNG release assay and the total response to CST (Supplementary File 6a,b) were not affected by OAF treatment.

## 4. Discussion

Administration of OAF did not affect BW, BCS, or milk yield in our experiment, confirming previous results [18,20]. Furthermore, the lack of differences in milk composition suggests OAF to have no direct effects on metabolic functions under homeostasis condition. Nevertheless, the lower NEFA and BHB concentration found in IMS animals after calving suggests OAF to have reduced lipomobilization and hepatic ketogenesis. Such a positive effect on lipid metabolism could be directly induced by niacin and choline contained in OAF. Niacin reduces hepatic lipolysis, and choline reduces triglyceride abundance and fat infiltration in the liver [39]; supplementation of these two compounds during the transition period has been previously reported to reduce blood NEFA and BHB concentrations in dairy cows [40,41].

The higher rumination time found in IMS cows in early lactation suggests OAF improved the recovery of rumen motility after the severe digestive and metabolic challenges that are widely reported during transition period [42,43]. This could be consequential to the modulatory effect that the yeasts and fungal cell walls composing OAF exert on rumen fermentation; however, the collection time of our rumen samples makes it difficult to elucidate the effect of the additive on rumen fermentation after the sudden alterations related to calving. Our samples were collected before morning feeding at 28 DFC, long after the occurrence of alterations related to calving and when fermentation patterns were at their nadir. Thus, the lack of any increase in VFA production with OAF does not dismiss its role in modulating rumen fermentation because such higher production could have been balanced by higher absorption. The amelioration in lipid metabolism with OAF could be hypothesized to partially contribute to the improved rumination time. In fact, 3 out of 5 of our CTR cows developed sub-clinical ketosis in early lactation (blood BHB > 1.4 mmol/L), and the higher rumination time found in our IMS cattle could be consequential to the inhibitory effect of ketosis on rumination activity [44] in our CTR cows. Despite that, the number of animals included in our experiment and the low statistical power found for rumination time measurements suggests care in the interpretation of such a founding.

The improved rumination activity and lipid metabolism in early lactation could account for the effectiveness of OAF in favoring the recovery of normal body functions after stress [6], suggesting it to be effective in preventing the metabolic disorders of TP. These positive effects on lipid metabolism and rumination could also account for the effectiveness of OAF in improving leukocyte functions [45,46,47]. Both NEFA and BHB are known to impair the viability of white blood cells [1,48,49], and their reduction could explain the lower lactate production and higher glucose concentration found in the blood of IMS cows after LPS stimulation of leukocytes. Production of lactate by neutrophils and monocytes is known to impair the motility, killing capacity and effector functions of leukocytes and suppressing the inflammasome and the production of pro-inflammatory cytokines [1,50]. Thus, a lower production of this metabolite in activated leukocytes could reflect greater immune competence. The lower glucose consumption after stimulation with LPS could be driven by more efficient metabolism in leukocytes while they cope with a biological stressor, reflecting improved killing capacity of those cells. This is also consistent with the previously reported effectiveness of OAF in improving phagocytosis, ROM production, and respiratory burst activity of PMN [19].

With these positive effects on lipomobilization and white blood cell function, an improvement in liver function and faster resolution of inflammatory phenomena, reflected in the typical trends of plasma indicators [51], were expected at a metabolic level with OAF supplementation. Nevertheless, the circulating concentration of IL-1B and IL-6 and their level after the ex vivo stimulation of leukocytes with LPS were not affected by the treatment, even though the augmented expression of genes triggered by toll-like receptors in PMNs has been widely reported as a main effect of OAF [6]. Furthermore, OAF did not affect the blood concentration of α-globulins (haptoglobin, ceruloplasmin, and SAA), called positive APP because their hepatic synthesis increases during inflammation [52]. Less clear is the effect of OAF on albumin, PON, and lipoprotein concentrations. These indicators are called negative APP because they decrease during inflammation due to the shift of liver synthesis on positive APP [53].

Our IMS animals tended to have a higher concentration of cholesterol in the early-dry period, reflecting greater hepatic synthesis of lipoproteins [54] and a better liver condition. A possible explanation for such an effect of OAF could be the contribution of vitamin B12 in increasing phospholipid concentrations in the blood [55]. The tendency for lower blood ROMt concentrations suggests lower production of oxidant species in our IMS cows during the dry period, which might have arisen from a less marked leukocyte activation in the peripheral blood, as suggested by the reduced myeloperoxidase and AOPP concentrations detected in the same time frame. In fact, myeloperoxidase generates hypochlorous acid that is converted into ROM within the respiratory burst [15,56], and AOPP are markers of protein oxidation triggered by this metabolite [57]. Such an anti-oxinflammatory effect exerted by OAF before calving could arise from better stabilization of PMN and monocytes, which need a stronger stimulus to mount an inflammatory reaction in IMS animals. Furthermore, the tendency for a lower production of ROM could suggest a more efficient oxidative phosphorylation pathway, in accordance with [6].

On the other hand, our IMS animals had lower levels of albumin, SHp, and FRAP in early lactation in comparison to CTR cows. These compounds are known to exert antioxidant activities: Albumin has a role in ligand binding and free radical trapping [58]; blood SHp mainly reflects albumin, lipoic acid, glutathione, and cysteine concentration; and FRAP provides a measurement of antioxidant power via blood concentration of bilirubin, uric acid, proteins, and vitamins C and E [59]. As antioxidant capacity directly depends on liver activity [15,60], dysregulation of the liver functions involved in controlling blood concentrations of such compounds could account for their post-partum trends with OAF; however, the lack of an effect on the concentration of bilirubin or enzymes related to amino acid metabolism in the liver (AST-GOT, GGT, ALP) refute the occurrence of any impairment of liver function [61]. Thus, we may hypothesize that the depletion of antioxidant systems observed with OAF in early lactation could arise from weak antioxidant activity of the product. Antioxidant biosynthesis and release are regulated processes, and administration of weak oxidants is known to up-regulate endogenous antioxidant mechanisms [62]. Conversely, weak antioxidant activity exerted by OAF could have reduced requirements for endogenous antioxidant compounds, down-regulating their synthesis de-novo.

## 5. Conclusions

Administration of OAF during the dry period and into lactation did not affect BW, BCS, milk yield, or milk and rumen fluid composition, nor did it affect peripheral response to the CST, reflecting neutrophil diapedesis. Nevertheless, OAF increased the rumination time and reduced NEFA and BHB concentrations in the blood in early lactation. Such an effect could explain the greater efficiency of leukocytes in facing biological stressors during the peripartum, as suggested by the lower lactate production and lower glucose consumption of leukocytes after an LPS challenge. Despite these positive effects on immune cells, OAF appears ineffective in reducing the degree of inflammation resulting from calving. A reduced abundance of antioxidants (albumin, SHp, and FRAP) also occurred with OAF after calving, suggesting a weak antioxidant action of the product reduced bodily requirements for endogenous antioxidant synthesis, although the lack of any effect on the ROM/FRAP ratio suggests no effect on the oxidative stress status. Although no effect was detected on liver function and inflammation, positive effects of OAF in reducing lipomobilization, favoring the recovery of rumen functions and exerting a probable antioxidant effect in early lactation suggest it to be an effective strategy in attenuating metabolic disorders of the transition period.

The associations identified here in a small number of cows in one herd should be further investigated. In fact, the evaluation of OAF on a larger number of animals and during acute inflammatory events is required to fully elucidate its effects on inflammatory parameters and leukocytes function. Furthermore, a deeper investigation of rumen fluid composition, with collection of rumen samples immediately after calving and when rumen fermentation reaches its peak, is required to elucidate how the effect of OAF on rumen level could contribute to ameliorating metabolic conditions in early lactation.

## Figures and Tables

**Table 1:** Composition and characteristics of the experimental diets fed to cows during the 5 experimental phases.

Diet, % DM	Phase 1 ^4^	Phase 2	Phase 3	Phase 4	Phase 5
DFC ^3^	−62 to −55	−47 to −7	−6 to 0	0 to 30	31 to 42
**Item**					
Corn silage	28.5	18.6	23.6	24.0	23.3
Alfalfa hay	16.4	-	-	13.7	11.1
Grass hay	23.4	71.4	58.0	18.1	11.9
Concentrate (−62 to 0 DFC ^3^)	-	10.0	10.1	5	-
Concentrate (1 to 42 DFC ^3^)	31.7	-	8.3	44.2	53.7
**Concentrate Composition, % DM**					
Corn flour	-	-	-	40.0	40.0
Barley flour	-	-	-	1.4	1.4
Sorghum grain expanded	-	-	-	-	-
Soybean meal	90.5	90.5	90.5	13.1	13.1
Soybean dry rolled	-	-	-	-	-
Sunflower meal	-	-	-	4.9	4.9
Corn gluten feed	-	-	-	-	-
Beet pulp	-	-	-	16.6	16.6
Wheat bran	-	-	-	9.8	9.8
Beet molasse slops	-	-	-	2.6	2.6
Potato protein	-	-	-	2.2	2.2
Hydrogenated palm oil	-	-	-	3.3	3.3
Limestone	-	-	-	1.39	1.39
Dicalcium phosphate	-	-	-	1.80	1.80
Sodium bicarbonate	-	-	-	0.98	0.98
Magnesium oxide	2.2	2.2	2.2	0.64	0.64
Sodium Chloride	1.4	1.4	1.4	0.32	0.32
Supplement ^1^	5.9	5.9	5.9	1.07	1.07
**Chemical Composition**					
NE_L,_ Mcal kg of DM^−1^	1.59	1.45	1.53	1.60	1.63
Crude protein, % DM	14.9	13.6	14.5	16.2	17.2
Starch + sugar, % DM	23.7	16.8	19.3	26.0	18.3
Ether extract, % DM	3.80	1.80	2.40	4.48	5.08
NDF, % DM	39.4	49.3	45.5	35.7	32.6
MP ^2^, % CP	9.8	9.1	9.7	10.5	11.1
RUP ^2^, % DM	4.64	4.48	4.77	5.23	5.96

^1^ Supplements were composited to provide 150,000 IU of vitamin A, 10,000 IU of vitamin D, 200 mg of vitamin E, 100 mg of vitamin K, 100 mg of vitamin H1, 50 mg of vitamin B1, 0.5 mg of vitamin B12, 500 mg of vitamin PP, 4000 mg of choline, 350 mg of Mn, 800 mg of Zn, 40 mg of Cu, 20 mg of I, 1 mg of Co, 1 mg Se. ^2^ Estimated using NRC 2001. ^3^ Days from calving. ^4^ Between −55 and −48 days from calving cows received only grass hay.

**Table 2:** Milk composition, rheological measurements, and somatic cell count in control dairy cows (n = 5) or cows receiving 65 g*d^−1^ of Omnigen-AF^®^ as top-dress between −55 and 42 days from calving (n = 5).

Item, Unit	TRT ^1^	Days from Calving				SE ^5^	*p*-Value		
		7	14	28	42		TRT ^1^	t ^2^	TRT × t ^3^
Butterfat, mg 100 mL^−1^	CTR	4.69	4.24	4.68	3.67	0.35	0.21	0.01	0.78
	IMS	4.33	4.14	3.82	3.36				
Fat output, kg	CTR	0.38	0.41	0.65	0.48	0.07	0.17	0.02	0.65
	IMS	0.27	0.37	0.48	0.44				
Total protein, mg 100 mL^−1^	CTR	3.84	3.30	3.07	3.09	0.10	0.49	<0.01	0.66
	IMS	3.81	3.34	3.25	3.19				
Protein output, kg	CTR	1.21	1.18	1.33	1.36	0.06	0.61	<0.01	0.15
	IMS	1.15	1.18	1.38	1.50				
Fat/protein ratio, -	CTR	1.22	1.28	1.54	1.19	0.12	0.11	0.11	0.64
	IMS	1.14	1.24	1.19	1.05				
Lactose, mg 100 mL^−1^	CTR	4.79	5.25	5.06	5.17	0.07	0.73	<0.01	0.03
	IMS	4.96	5.09	5.16	5.14				
		+							
Caseins, mg 100 mL^−1^	CTR	2.84	2.50	2.28	2.33	0.08	0.42	<0.01	0.59
	IMS	2.85	2.51	2.45	2.40				
Titratable acidity, °SH 50 mL^−1^	CTR	3.96	3.33	3.23	3.12	0.17	0.77	<0.01	0.93
	IMS	3.86	3.19	3.20	3.12				
Urea-N, mg dL^−1^	CTR	31.2	34.7	26.8	31.2	3.11	0.64	0.08	0.66
	IMS	33.7	33.3	30.2	33.0				
Coagulation time (rCT), min	CTR	12.3	20.0	14.0	14.5	2.03	0.77	<0.01	0.68
	IMS	11.5	16.2	14.8	16.1				
Curd firmness (a30), mm	CTR	37.7	23.2	35.3	32.8	4.59	0.27	0.31	0.40
	IMS	43.4	33.6	35.6	34.7				
SCC ^4^, 10^3^ mL^−1^	CTR	293.4	-	37.7	-	91.4	0.47	0.18	0.31
	IMS	72.4	-	54.9	-				

^1^ Treatment (CTR is control cows; IMS is cows receiving a topdressing of the feeds with the immunostimulant Omnigen-AF^®^). ^2^ Time. ^3^ Treatment × time interaction (+ is *p* < 0.10 for differences among means within a column. This symbol is only presented when the interaction effect is significant). ^4^ Somatic cells count. ^5^ Standard error = larger standard error for the fixed effects.

**Table 3:** Ruminal pH, ammonia concentrations and volatile fatty acids molar proportion, determined at 28 days after calving in control dairy cows (n = 5) or cows receiving 65 g*d^−1^ of Omnigen-AF^®^ as top-dress between −55 and 42 days from calving (n = 5).

Item	Unit	TRT ^1^		SE ^3^	*p*-Value
		CTR	IMS		
pH	-	6.78	6.54	0.15	0.14
Ammonia-N	mg L^−1^	4.65	4.47	0.10	0.96
Total VFA ^2^	m*M* L^−1^	103.8	109.0	8.72	0.52
Individual VFA	mol 100 mol^−1^ total VFA				
Acetic acid		66.2	67.0	5.08	0.79
Propionic acid		21.8	25.4	3.81	0.38
Butyric acid		11.4	12.3	1.17	0.47
Isobutyric acid		0.89	0.80	0.08	0.39
Valeric acid		1.26	1.42	0.21	0.41
Isovaleric acid		1.55	1.57	0.14	0.77
Caproic acid		0.55	0.46	0.14	0.61
Enanthic acid		0.04	0.03	0.01	0.35
L-lactic acid	m*M* L^−1^	0.39	0.37	0.11	0.88
D-lactic acid	m*M* L^−1^	0.39	0.37	0.11	0.91
Total lactic acid	m*M* L^−1^	0.78	0.75	0.21	0.89

^1^ Treatment (CTR is control cows; IMS is cows receiving a topdressing of the feeds with the immunostimulant Omnigen-AF^®^). ^2^ Total volatile fatty acids. ^3^ Standard error = larger standard error for the fixed effects.

**Table 4:** Blood cells profile in control dairy cows (n = 5) or cows receiving 65 g*d^−1^ of Omnigen-AF^®^ as top-dress between −55 and 42 days from calving (n = 5).

Item, Unit	TRT ^1^	Days from Calving						SE ^2^	*p*-Value		
		−62	−48	−21	−3	7	28		TRT^1^	t ^3^	TRT × t ^4^
WBC ^5^, 10^3^ µL^−1^	CTR	6.4	5.8	6.3	7.7	6.0	5.9	0.77	0.54	<0.01	0.59
	IMS	7.5	6.6	6.8	8.4	6.8	5.7	0.77			
Neu ^6^, 10^3^ µL^−1^	CTR	3.3	3.0	3.2	4.7	3.6	3.2	0.61	0.52	<0.01	0.69
	IMS	4.0	3.3	3.5	5.1	3.9	3.0	0.61			
Lym ^7^, 10^3^ µL^−1^	CTR	2.2	2.0	2.2	2.2	1.7	1.9	0.59	0.56	<0.01	0.04
	IMS	2.7	2.6	2.6	2.3	2.2	2.0	0.61			
HGB ^8^, g dL^−1^	CTR	10.8	11.4	10.6	11.0	10.7	9.8	0.40	0.20	<0.01	0.42
	IMS	10.0	10.5	10.0	10.2	10.4	9.4	0.40			
MCV ^9^, fL	CTR	48.3	48.6	48.6	51.0	49.6	48.7	1.4	0.08	<0.01	0.78
	IMS	45.8	45.5	45.9	47.5	47.6	46.8	1.4			
MCH ^10^, pg	CTR	16.5	16.9	16.9	17.2	17.1	16.9	0.47	0.08	0.06	0.69
	IMS	15.7	15.7	16.0	16.5	16.4	16.4	0.47			
PLT ^11^, 10^3^ µL^−1^	CTR	323.8	273.6	280.0	305.8	313.8	420.4	38.2	0.07	0.02	0.84
	IMS	354.6	348.8	345.8	412.8	357.2	437.6	38.2			
MPV ^12^, fL	CTR	6.9	8.3	7.6	6.9	6.8	5.8	0.36	0.01	0.01	0.01
	IMS	6.1	5.7	6.3	6.5	5.9	5.5	0.45			
			**	*		+					

^1^ Treatment (CTR is control cows; IMS is cows receiving a topdressing of the feeds with the immunostimulant Omnigen-AF^®^). ^2^ Standard error = larger standard error for the fixed effects. ^3^ Time effect. ^4^ Treatment × time interaction effect (+ is *p* < 0.10; * is *p* < 0.05; ** is *p* < 0.01 for differences among means within a column. These symbols are only presented when the interaction effect is significant). ^5^ White blood cells. ^6^ Neutrophils. ^7^ Lymphocytes. ^8^ Hemoglobin. ^9^ Mean cell volume. ^10^ Mean cell hemoglobin. ^11^ Platelets. ^12^ Mean platelet volume.

**Table 5:** Fold change values (expressed with respect to baseline) of cytokines in a whole blood stimulation assay with a low or high dose of bacterial lipopolysaccharides in control dairy cows (n = 5) or cows receiving 65 g*d^−1^ of Omnigen-AF^®^ as top-dress between −55 and 42 days from calving (n = 5).

Item	D ^2^	TRT ^1^	Days from Calving						SE ^3^	*p*-Value				
			−62	−45	−21	−3	7	28		TRT ^1^	t ^4^	D ^2^	TRT × t ^5^	TRT × t × D ^6^
IL-1 ^7^	Low	CTR	3.5	9.9	10.0	20.2	18.7	6.9	11.3	0.66	<0.01	<0.01	0.13	0.87
		IMS	5.2	9.9	13.1	10.2	9.8	9.7						
	High	CTR	13.1	21.2	33.6	52.1	55.3	23.7	11.3					
		IMS	20.0	40.9	39.9	51.8	59.6	32.3						
	Total	CTR	8.3	15.5	21.8	36.1	37.0	15.3	9.3					
		IMS	12.6	25.4	26.5	31.0	34.7	21.0						
IL-6 ^8^	Low	CTR	1.34	1.61	1.27	1.92	1.57	1.91	0.39	0.97	0.06	<0.01	0.10	0.95
		IMS	1.69	1.65	1.56	1.33	1.78	1.60						
	High	CTR	1.90	2.19	1.99	2.10	2.40	3.07	0.39					
		IMS	2.20	2.38	1.77	1.71	2.87	2.38						
	Total	CTR	1.62	1.90	1.63	2.01	1.98	2.49	0.31					
		IMS	1.95	2.01	1.67	1.52	2.32	1.99						

^1^ Treatment (CTR is control cows; IMS is cows receiving a topdressing of the feeds with the immunostimulant Omnigen-AF^®^). ^2^ Dose (Total reflects the global effect of LPS stimulation, without accounting for the dose). ^3^ Standard error = larger standard error for the fixed effects. ^4^ Time. ^5^ Treatment × time interaction effect. ^6^ Treatment × time × dose interaction effect. ^7^ Interleukin-1 beta. ^8^ Interleukin-6.

**Table 6:** Fold change values (expressed with respect to baseline) of energy metabolism biomarkers after a whole blood stimulation assay with a low or high dose of bacterial lipopolysaccharides in control dairy cows (n = 5) or cows receiving 65 g*d^−1^ of Omnigen-AF^®^ as top-dress between −55 and 42 days from calving (n = 5).

Item	D ^2^	TRT ^1^	Days from Calving						SE ^3^	*p*-Value				
			−62	−45	−21	−3	7	28		TRT ^1^	t ^4^	D ^2^	TRT × t ^5^	TRT × t × D ^6^
Glu ^7^	Low	CTR	0.96	0.96	0.94	0.96	0.93	0.97	0.04	0.13	0.41	0.02	0.06	1.00
		IMS	0.98	0.98	0.99	0.91	1.08	0.97						
	High	CTR	0.95	0.94	0.89	0.91	0.88	0.93	0.04					
		IMS	0.92	0.93	0.96	0.85	1.00	0.91						
	Total	CTR	0.95	0.95	0.91	0.93	0.91	0.95	0.03					
		IMS	0.95	0.95	0.98	0.88	1.04	0.94						
					+		**							
DLA ^8^	Low	CTR	0.97	1.00	1.05	1.12	1.08	1.09	0.04	0.62	0.01	0.04	0.08	0.99
		IMS	1.02	1.03	0.99	1.07	1.01	0.99						
	High	CTR	0.98	1.00	1.09	1.13	1.11	1.15	0.04					
		IMS	1.06	1.06	1.05	1.12	1.09	1.07						
	Total	CTR	0.98	1.00	1.07	1.12	1.10	1.12	0.03					
		IMS	1.04	1.05	1.02	1.10	1.05	1.03						
								+						
LLA ^9^	Low	CTR	1.02	1.02	1.02	1.04	1.06	1.06	0.02	0.35	0.10	<0.01	0.10	0.84
		IMS	1.04	1.02	1.03	1.04	1.02	1.00						
	High	CTR	1.03	1.03	1.08	1.07	1.10	1.14	0.02					
		IMS	1.07	1.03	1.04	1.06	1.05	1.08						
	Total	CTR	1.02	1.02	1.05	1.06	1.08	1.10	0.02					
		IMS	1.05	1.02	1.04	1.05	1.04	1.04						
								*						

^1^ Treatment (CTR is control cows; IMS is cows receiving a topdressing of the feeds with the immunostimulant Omnigen-AF^®^). ^2^ Dose (Total reflects the global effect of LPS stimulation, without accounting for the dose). ^3^ Standard error = larger standard error for the fixed effects. ^4^ Time. ^5^ Treatment × time interaction effect (+ is *p* < 0.10; * is *p* < 0.05; ** is *p* < 0.01 for differences among means within a column. These symbols are only presented when the interaction effect is significant). ^6^ Treatment × time × dose interaction effect. ^7^ Glucose. ^8^ D-lactic acid. ^9^ L-lactic acid.

**Table 7:** Fold change values (expressed with respect to baseline) of nitrogen species after a whole blood stimulation assay with a low or high dose of bacterial lipopolysaccharides in control dairy cows (n = 5) or cows receiving 65 g*d^−1^ of Omnigen-AF^®^ as top-dress between −55 and 42 days from calving (n = 5).

Item	D ^2^	TRT ^1^	Days from Calving						SE ^3^	*p*-Value				
			−62	−45	−21	−3	7	28		TRT ^1^	t ^4^	D ^2^	TRT × t ^5^	TRT × t × D ^6^
NO_2_^7^	Low	CTR	1.02	0.99	1.03	1.11	0.96	1.13	0.07	0.96	<0.01	0.74	0.16	0.70
		IMS	0.97	0.97	1.23	1.23	1.03	0.97						
	High	CTR	1.04	1.00	1.03	1.18	0.99	1.06	0.07					
		IMS	1.05	1.01	1.01	1.14	1.02	0.95						
	Total	CTR	1.03	1.00	1.03	1.15	0.97	1.10	0.05					
		IMS	1.01	0.99	1.12	1.18	1.03	0.96						
NO_3_^8^	Low	CTR	0.95	1.07	1.05	1.06	1.04	1.01	0.12	0.96	0.99	0.03	0.94	0.78
		IMS	0.99	1.01	0.98	1.02	1.02	0.96						
	High	CTR	1.20	1.05	1.20	1.05	1.04	1.05	0.12					
		IMS	1.14	1.40	1.13	1.02	1.11	1.23						
	Total	CTR	1.07	1.06	1.12	1.05	1.04	1.03	0.08					
		IMS	1.06	1.20	1.05	1.02	1.07	1.09						
NOx ^9^	Low	CTR	0.98	1.05	1.05	1.07	1.02	1.05	0.07	0.73	0.97	0.03	0.94	0.79
		IMS	0.98	1.01	1.03	1.05	1.03	0.97						
	High	CTR	1.14	1.08	1.15	1.08	1.02	1.05	0.07					
		IMS	1.11	1.17	1.07	1.04	1.09	1.14						
	Total	CTR	1.06	1.06	1.10	1.08	1.02	1.05	0.05					
		IMS	1.05	1.09	1.05	1.04	1.06	1.06						

^1^ Treatment (CTR is control cows; IMS is cows receiving a topdressing of the feeds with the immunostimulant Omnigen-AF^®^). ^2^ Dose (Total reflects the global effect of LPS stimulation, without accounting for the dose). ^3^ Standard error = larger standard error for the fixed effects. ^4^ Time. ^5^ Treatment × time interaction effect. ^6^ Treatment × time × dose interaction effect. ^7^ Nitrite. ^8^ Nitrate. ^9^ Nitric oxide.

## Supplementary Materials

The following are available online at https://www.mdpi.com/2076-2615/9/9/619/s1.
