# Supplementary material for: Administration of an Immune Stimulant during the Transition Period Improved Lipid Metabolism and Rumination without Affecting Inflammatory Status
Source: Animals (Basel). 2019 Aug 28;9(9):619. doi: 10.3390/ani9090619 (PMC6770279; doi:10.3390/ani9090619)
Supplement: Supplementary file 1 [file animals-09-00619-s001.pdf]

Supplementary File 1. Intra- and inter-assay coefficient of variations, limit of quantification (LOQ), codes of commercial kits used, references for their validation in the bovine plasma, calibrators and quality controls used for plasma parameters included in the study.

Parameter	Inter	Intra	LOQ	Kit	Reference	Calibrator	Quality control
Glucose	0.75	1.33	0.10	0018250840[1]	-	Homemade bovine standard	Homemade bovine standard; SeraChem Control Level 1, 0018162412[1]; Bov Asy Control 2, AN1026[3]
Cholesterol	1.38	1.56	0.10	0018250540[1]	-		
Urea	1.23	1.14	0.01	0018255440[1]	-		
Calcium	1.86	0.93	0.20	0018250340[1]	-		
Phosphorus	0.73	1.64	0.01	0018251240[1]	-		
Magnesium	1.65	1.00	0.02	0018259240[1]	-		
Total proteins	2.87	1.07	10.00	00182514401	-		
Albumins	3.41	1.20	16.00	00182500401	-		
Creatinine	3.09	1.09	18.00	00182555401	-		
Sodium	1.53	0.62	50.00	ISE Diluent, 0018253400[1]; ISE Reference, 0018253500[1]	-	ISE low calibrator, 0018469200[1]; ISE high calibrator, 0018469300[1]	
Potassium	1.11	0.63	1.46		-		
Chlorine	0.77	0.34	70.00		-		
Zinc	3.83	1.76	0.500	439-14906[2]	-	Bov Asy Control 2[4]	
Ceruloplasmin	3.57	1.39	0.100	-	[63]	Human plasma ceruloplasmin, A50143H[6]	
AST / GOT	3.55	1.78	1.00	0018257540[1]	-	No calibration is required	
GGT	4.42	2.27	2.00	0018257640[1]	-		
Paraoxonase	4.60	1.38	1.20	-	[64]		
Haptoglobin	3.50	3.64	0.01	-	[65]		
Total Bilirubin	2.46	3.11	2.00	0018254640[1]	-	Bov Asy Control 2[4]	
Alkaline phosphatase	3.52	0.77	2.00	0018259640[1]	-	ReferrIL E, 0018256300[1]	
NEFA	5.00	2.27	0.010	NEFA-HR(2) R1 Set, 434-91795[2]	-	NEFA standard, 270-77000[2]	
BHB	3.42	0.82	0.100	RB1007[3]	-	BHB standard included in the kit	
AOPP	10.93	0.42	10.00	-	[33]	Chloramine T trihydrate, 31224-250G [10]	
ROMt	2.93	1.40	3.20	MC003[4]	-	Calibrator d-ROMs, MC.030[9]	Homemade bovine standard SeraChem Control Level 1, 0018162412[1]; Control Serum, MC031[4]; Calibrator d-ROMs, MC030[4]
FRAP antioxidants	3.15	3.13	30.00	-	[59]	TROLOX, 238813-1G[8]	Homemade bovine standard; SeraChem Control Level 1, 0018162412[1]; Bov Asy Control 2, AN1026[3]; TROLOX, 238813-1G[8]
D-lactic acid	2.30	1.20	0,214	K-DLATE[5]	-	No calibration is required	D-/L- Lactic acid standard included in the kit
L-lactic Acid	2.10	1.20	0,214		-		
NO2	7.55	1.58	1.20	-	[66,67]	NaNO2, 481827[9]	Homemade bovine standard;
NOx	7.32	1.68	8.00				

							SeraChem Control Level 1, 0018162412[1]; Bov Asy Control 2, AN1026[3]; NaNO2 solution
Myeloperoxidase	8.60	1.83	10.00	-	[32]	No calibration is required	Homemade bovine standard; SeraChem Control Level 1, 0018162412[1]
IL6	15.57	8.60	78.00	ESS0029[7]	-	Bovine IL6 standard included in the kit	Homemade bovine standard
IL1B	16.20	5.90	15.00	ESS0027[7]	-	Bovine IL1B standard included in the kit	

[1]Instrumentation Laboratory Werfen, Milano, Italy. [2]Wako Chemicals GmbH, Neuss, Germany. [3]Randox Laboratories Ltd., Crumlin, County Antrim, UK. [4]Diacron International S.r.l., Grosseto, Italy. [5]Megazyme, Wicklow, Ireland. [6]Meridian Life Science, Memphis, USA. [7]Thermo Scientific, Frederick, MD 2174, USA. [8]Sigma-Aldrich Chemie GmbH, Steinheim, Germany. [9]Carlo Erba, Rodano, Milano, Italy. [10]Sigma Aldrich, St. Louis, MO63103 USA.

Supplementary file 2. Trends of body weight (BW) and body condition score (BCS) in control dairy cows (n = 5) or cows receiving 65 g*d-1 of Omnigen-AF® as top-dress between -55 and 42 days from calving (n = 5).

Item, Unit	TRT[1]	Days from calving								P-Value		
		-65	-45	-21	1	14	21	28	45	TRT[1]	t[3]	TRT X t[4]
BW, kg	CTR	644.6	649.8	683.6	630.6	587.6	582.2	582.9	585.9	0.96	<0.01	0.99
	IMS	638.2	643.2	679.0	631.4	593.5	585.4	583.8	578.5			
BCS, -	CTR	2.55	2.54	2.56	2.51	2.28	2.20	2.19	2.15	0.54	<0.01	0.88
	IMS	2.41	2.43	2.45	2.44	2.26	2.13	2.13	2.04			
ΔBCS[2], -	CTR				0.32					0.94	-	-
	IMS				0.31							

[1]Treatment (CTR is control cows; IMS is cows receiving a topdressing of the feeds with the immunostimulant. [2]Difference between BCS value at calving and 28 DFC. [3]Time. [4]Treatment X time interaction effect.

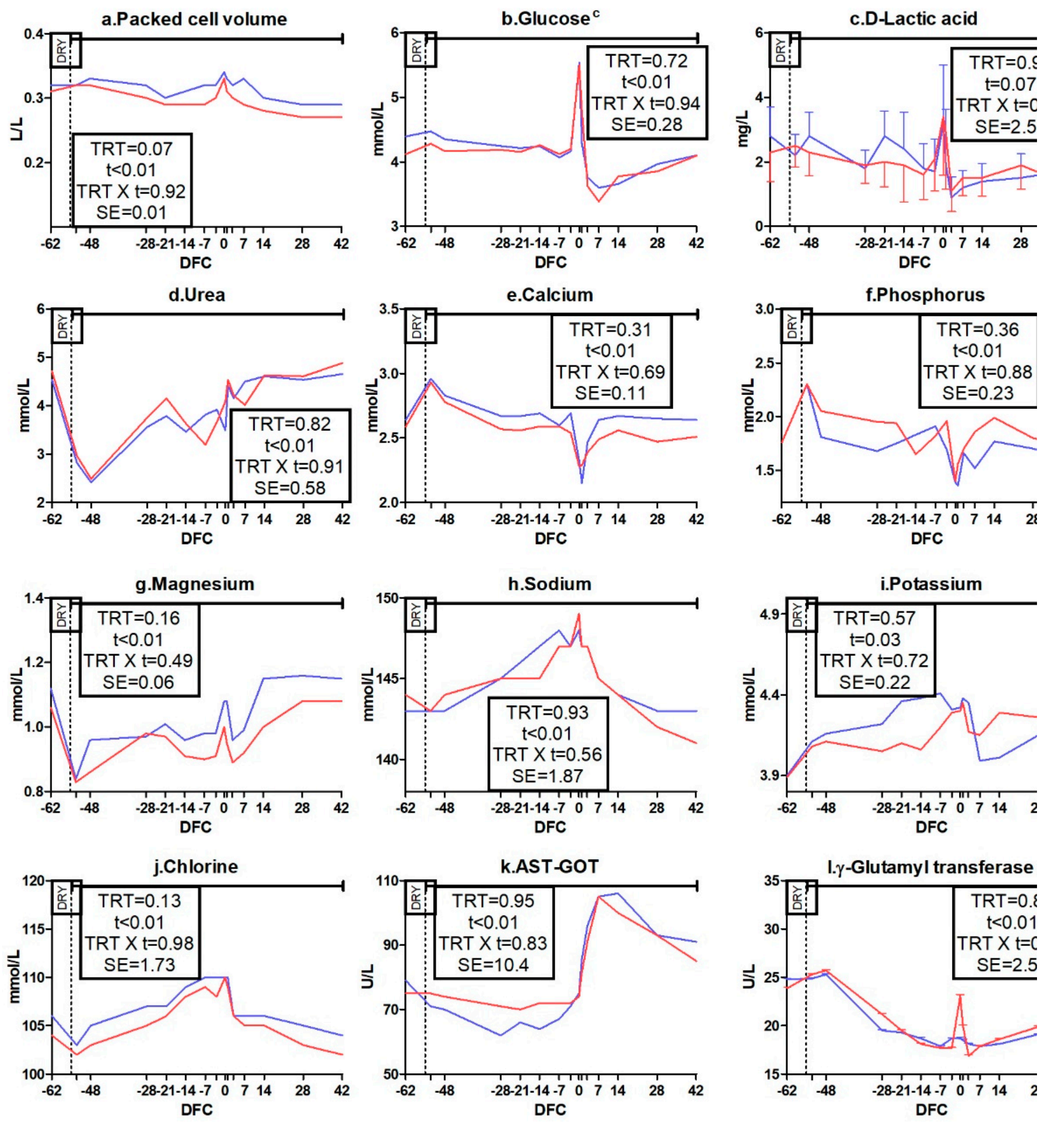

Supplementary file 3. Time course of packed cell volume (a) and plasma concentrations of glucose (b), D-lactic acid (c), urea (d), calcium (e), phosphorus (f), magnesium (g), sodium (h), potassium (i), chlorine (j), aspartate aminotransferase – glutamate oxaloacetate transaminase (AST-GOT; k) and γ-glutamyl transferase (l) in control dairy cows (CTR; blue line, n = 5) and cows receiving 65 g*d-1 of Omnigen-AF as top-dress (IMS; red line, n = 5) between -55 and 42 days from calving. Upper solid line indicates timing of treatment administration. TRT is treatment effect; t is time effect; TRT X t is the treatment X time interaction effect. DFC is days from calving; DRY is dry-off day (-55 days from expected calving). SE is standard error. [c]Parameter was covariate on -62 values.

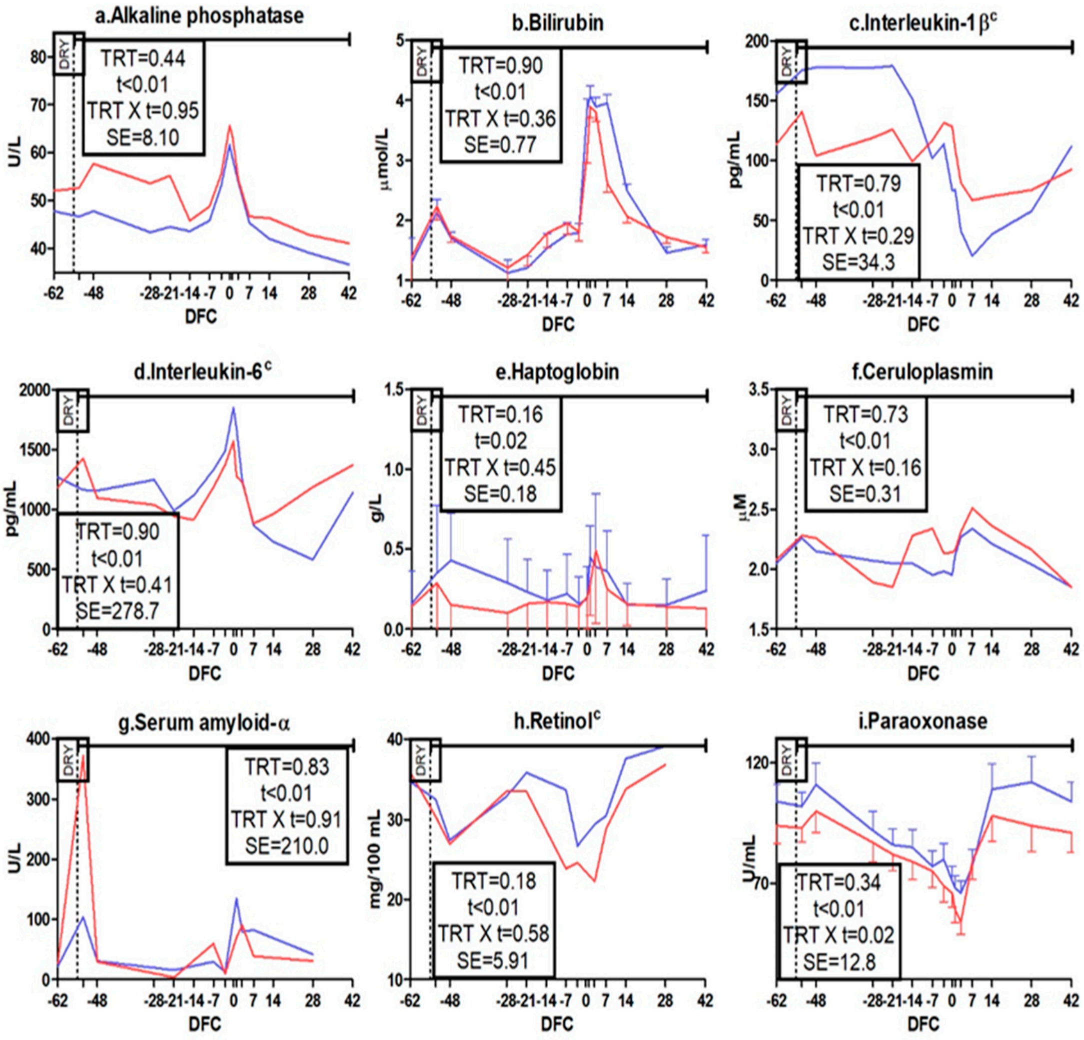

Supplementary file 4. Time course of plasma concentrations of alkaline phosphatase (a), bilirubin (b), interleukin-1 beta (c), interleukin-6 (d), haptoglobin (e), ceruloplasmin (f), serum amyloid-*α* (g), retinol (h) and paraoxonase (i) in control dairy cows (CTR; blue line, n = 5) and cows receiving 65 g*d-1 of Omnigen-AF as top-dress (IMS; red line, n = 5) between -55 and 42 days from calving. Upper solid line indicates timing of treatment administration. TRT is treatment effect; t is time effect; TRT X t is the treatment X time interaction effect. DFC is days from calving; DRY is dry-off day (-55 days from expected calving). SE is standard error. [c]Parameter was covariate on -62 values.

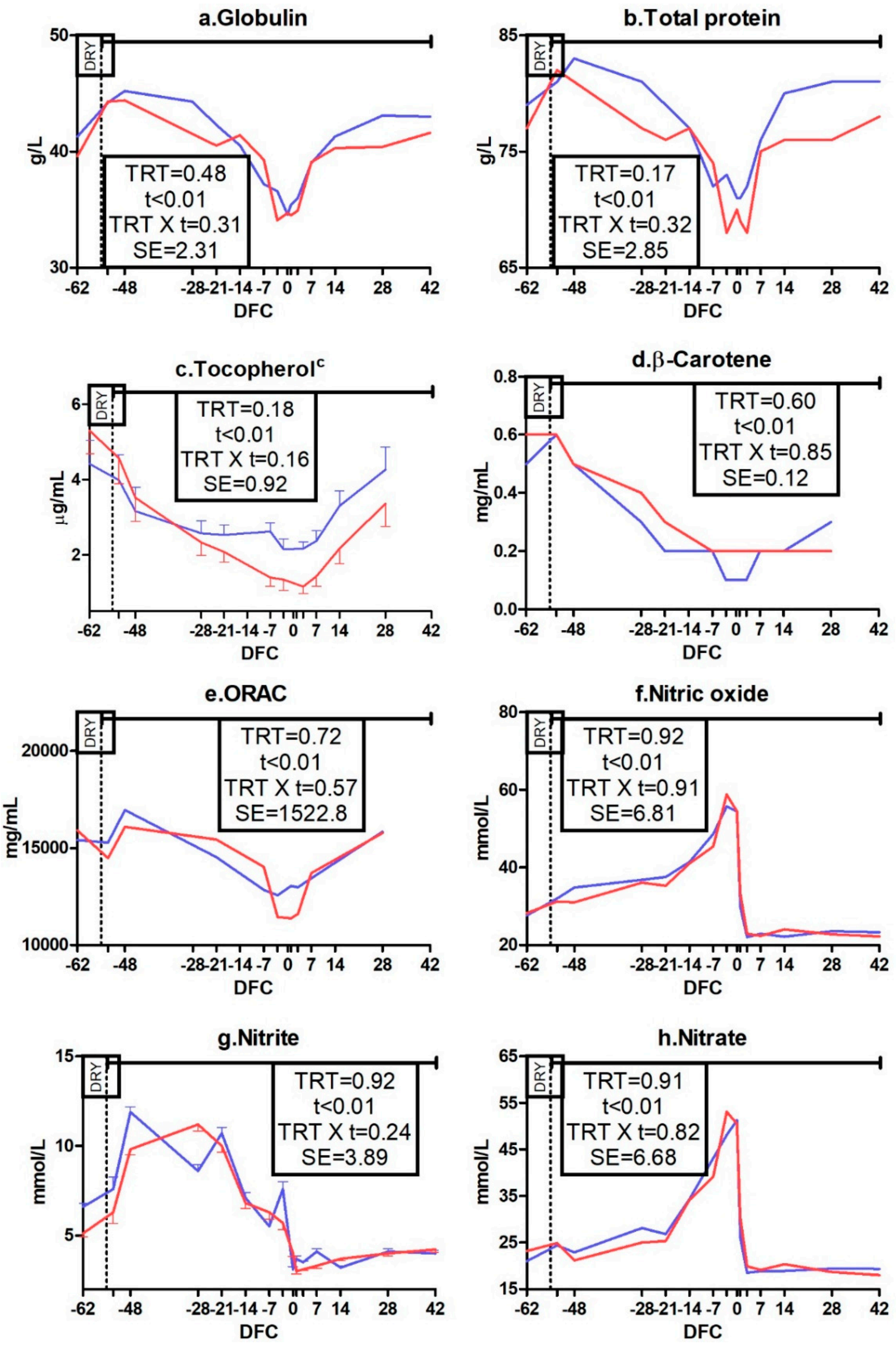

Supplementary file 5. Time course of plasma concentrations of globulin (a), total protein (b), tocopherol (c), β-carotene (d), oxygen reactive antioxidant capacity (ORAC; e), nitric oxide (f), nitrite (g) and nitrate (h) in control dairy cows (CTR; solid line, n = 5) and cows receiving 65 g*d-1 of Omnigen-AF as top-dress (IMS; dotted line, n = 5) between -55 and 42 days from calving. Upper solid line indicates timing of treatment administration. TRT is treatment effect; t is time effect; TRT X t is the treatment X time interaction effect. DFC is days from calving; DRY is dry-off day (-55 days from expected calving). SE is standard error; [c]Parameter was covariate on -62 values.

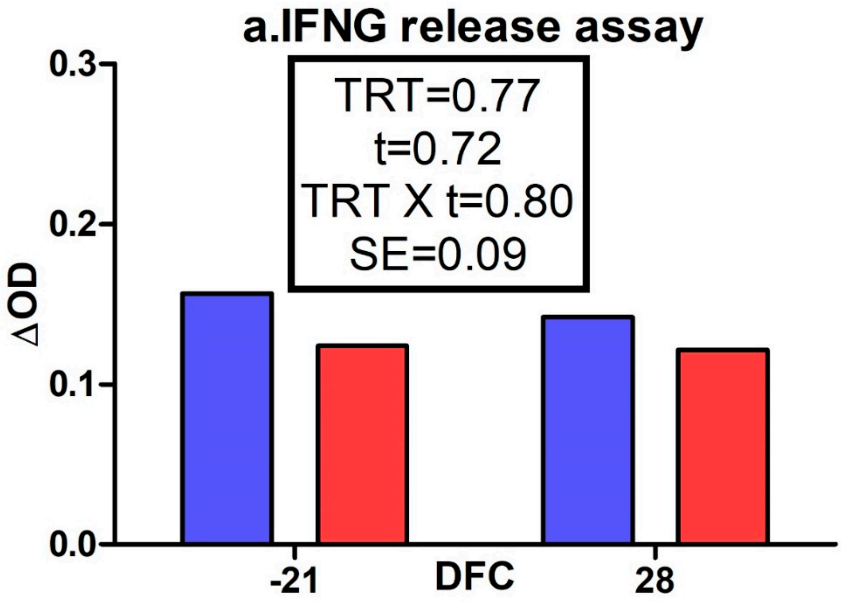

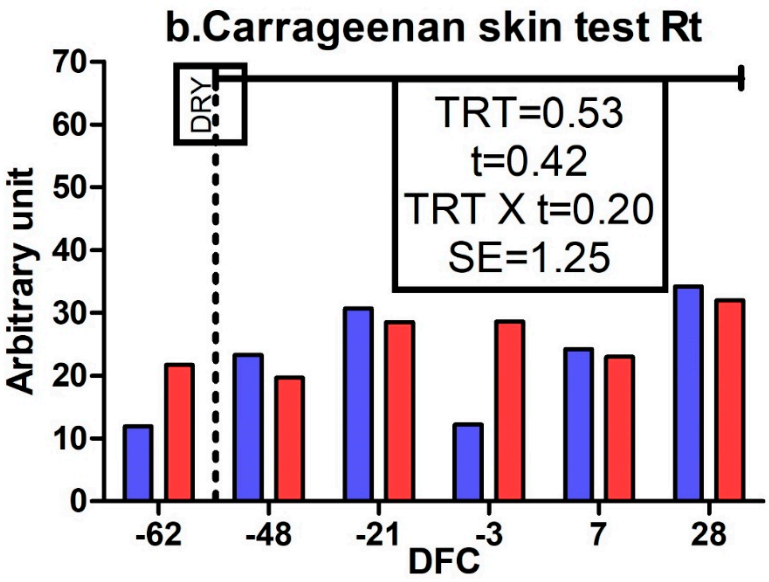

.

Supplementary file 6. Optical density difference (ΔOD) between avian PPD-stimulated and control wells in the interferon gamma (IFNG) release assay (a) and total peripheral response (Rt) after the carrageenan skin test (b) in control dairy cows (CTR; blue bars, n = 5) and cows receiving 65 g*d-1 of Omnigen-AF® as top-dress (IMS; red bars, n = 5) between -55 and 42 days from calving. Upper solid line indicates timing of treatment administration. TRT is treatment effect; t is time effect; TRT X t is the treatment X time interaction effect. DFC is days from calving; DRY is dry-off day (-55 days from expected calving). SE is standard error.
